# Seasonal patterns of faecal egg counts and gastrointestinal nematode species composition in Scottish dairy calves

**DOI:** 10.1016/j.vetpar.2025.110574

**Published:** 2025-08-07

**Authors:** Paul Campbell, Jennifer McIntyre, Kerry O’Neill, Andrew Forbes, Roz Laing, Kathryn Ellis

**Affiliations:** School of Biodiversity, One Health and Veterinary Medicine, University of Glasgow, Glasgow, UK

**Keywords:** Gastrointestinal nematodes, Dairy cattle, Species composition, Faecal egg counts, Anthelmintic treatment

## Abstract

Gastrointestinal nematode (GIN) infections impact livestock production globally. In pasture-based systems, GIN infections are ubiquitous, typically comprising co-infections with several different species within a single host. Nematode species vary in their epidemiology, pathogenicity, and anthelmintic sensitivity, which in turn can be influenced by weather, host factors, and management practices. The epidemiology of parasitic gastroenteritis in young cattle in temperate regions has been thoroughly researched. However, many studies were conducted more than fifty years ago, before the advent of modern molecular techniques and the widespread use of macrocyclic lactone (ML) anthelmintics. This study’s objective was to survey dairy farms with different management profiles, using faecal egg counts (FEC) and GIN L_3_ identification, to determine if any changes had occurred since these original studies. The longitudinal study of 23 Scottish dairy farms included 131 monthly sampling points, from which 1967 individual FECs were conducted, and a minimum of 94 L_3_ from pooled coprocultures identified by PCR (n = 13,297) per visit. Species composition and FEC followed expected patterns, yet varied considerably in relation to management and anthelmintic use; *Cooperia oncophora* was more abundant earlier in the grazing season, while *Ostertagia ostertagi* became more abundant as the season progressed. Other GIN observed included Trichostrongylus spp., Oesophagostomum spp., and *Haemonchus contortus*. The majority of farms relied entirely on ML products and had done so for many years. Farmer concerns regarding anthelmintic resistance were minimal, and few farms routinely employed FECs to aid management decisions. Regardless of treatment strategy, the groups exhibited no evidence of clinical disease, and FECs remained relatively low throughout, even on farms not using any anthelmintic treatment (0–480 eggs per gram).

## 1 Introduction

Infection with gastrointestinal nematode (GIN) species is ubiquitous in pasture-based livestock systems. In first-grazing-season (FGS) calves, the intensity of infection and species composition broadly determine the risk of clinical disease and the severity of subclinical production losses. First-grazing-season calves are naïve and particularly susceptible to such infections when grazing for the first time, typically occurring after weaning and within the first year of life. Parasitic gastroenteritis (PGE) in cattle is unlikely to cause mortality in UK systems, but it is a significant production-limiting infection, primarily due to the reduced growth rate in youngstock (Vercruysse and Claerebout, 2001). In the UK and other temperate regions, the most commonly observed GIN species infecting young cattle are *Ostertagia ostertagi* and *Cooperia oncophora*, located in the abomasum and small intestine, respectively. *Ostertagia ostertagi* is the more pathogenic species, but its impact can be increased when animals are co-infected with *C. oncophora* (Kloosterman et al., 1984; Parkins et al., 1990). The number and composition of a GIN nematode population are key determinants of their pathogenicity, the onset and development of immunity within the host (Vercruysse and Claerebout, 1997), and their propensity to tolerate or develop resistance to an anthelmintic (Coles, 2002). Many non-biological factors, such as climatic and environmental conditions, grazing and pasture management, and the age and diversity of hosts, will also influence the epidemiology of such infections (Armour, 1989; Forbes, 2017; Githigia et al., 2001; Stromberg, 1997). Scotland experiences a northern temperate climate, with an annual average temperature of 8.5 °C in 2022, characterised by relatively constant humidity and an average annual rainfall of 1574 mm (Kendon et al., 2023). The pasture grazing of dairy farms is primarily composed of improved grassland with high proportions of ryegrass-based swards; these pastures are often regularly fertilised with inorganic fertilisers, reseeded, and high-yielding.

Understanding the dynamics of GIN infections throughout the grazing season in different production systems and regions is a prerequisite for effective and sustainable parasite control. There have been many changes in dairy farming since the 1960s and 70 s, when many of the epidemiological studies were conducted, including cow genetics, milk yield, calving patterns, feeding regimes, pasture management, and, in terms of PGE, the introduction of the macrocyclic lactone parasiticides. In addition, factors such as climate change and the increasing threat of anthelmintic resistance could all influence the epidemiology and future control of GIN infections (Morgan et al., 2019; Skuce et al., 2013). Already, changing epidemiological patterns in the UK have been observed in *Nematodirus battus* in sheep and *Dictyocaulus viviparus* in cattle (McCarthy and van Dijk, 2020; Melville et al., 2021).

In the UK, limited recent research has examined the seasonal patterns of cattle GIN infection, leaving apparent gaps in our understanding. While previous studies have investigated the seasonal dynamics of GIN infections (Armour et al., 1979; Eysker and Van Miltenburg, 1988; Lancaster and Hong, 1987; Michel, 1969a, 1969b, 1969c; Rose, 1970), these historical studies relied on the morphological identification of infective larvae and were conducted before the advent of macrocyclic lactone products, which are now widely used in all livestock sectors. In contrast, extensive research has been conducted on sheep GIN, both historically and more recently, emphasising the prevalence of anthelmintic resistance (Rose Vineer et al., 2020a). Notably, Rose Vineer et al. (2020a) found that, in most European countries, the detected prevalence of anthelmintic resistance was positively correlated with research labour. The interactions between co-infecting parasitic helminth species are still poorly understood (Evans et al., 2023; Pedersen and Fenton, 2007), and assessment of species prevalence is a critical element of the host-parasite interaction. Mathematical modelling is increasingly being applied to parasitic helminth infections in livestock (Filipe et al., 2023; Rose et al., 2015; Rose Vineer et al., 2020b), and therefore, updated and comprehensive investigations are required, using modern molecular techniques applied to various management systems.

This study aimed to characterise the GIN infection patterns of a range of dairy calf management practices using modern molecular techniques and to generate data useful in guiding future research and parasite control initiatives. We compare the GIN communities and FEC patterns of calves throughout their first grazing season in 23 commercial Scottish dairy herds.

## 2 Materials and methods

The University of Glasgow MVLS College Ethics Committee (Project No: 200210097) approved all research procedures involving animal use.

### 2.1 Study design, farm recruitment, and selection criteria

**Fig. 1:**
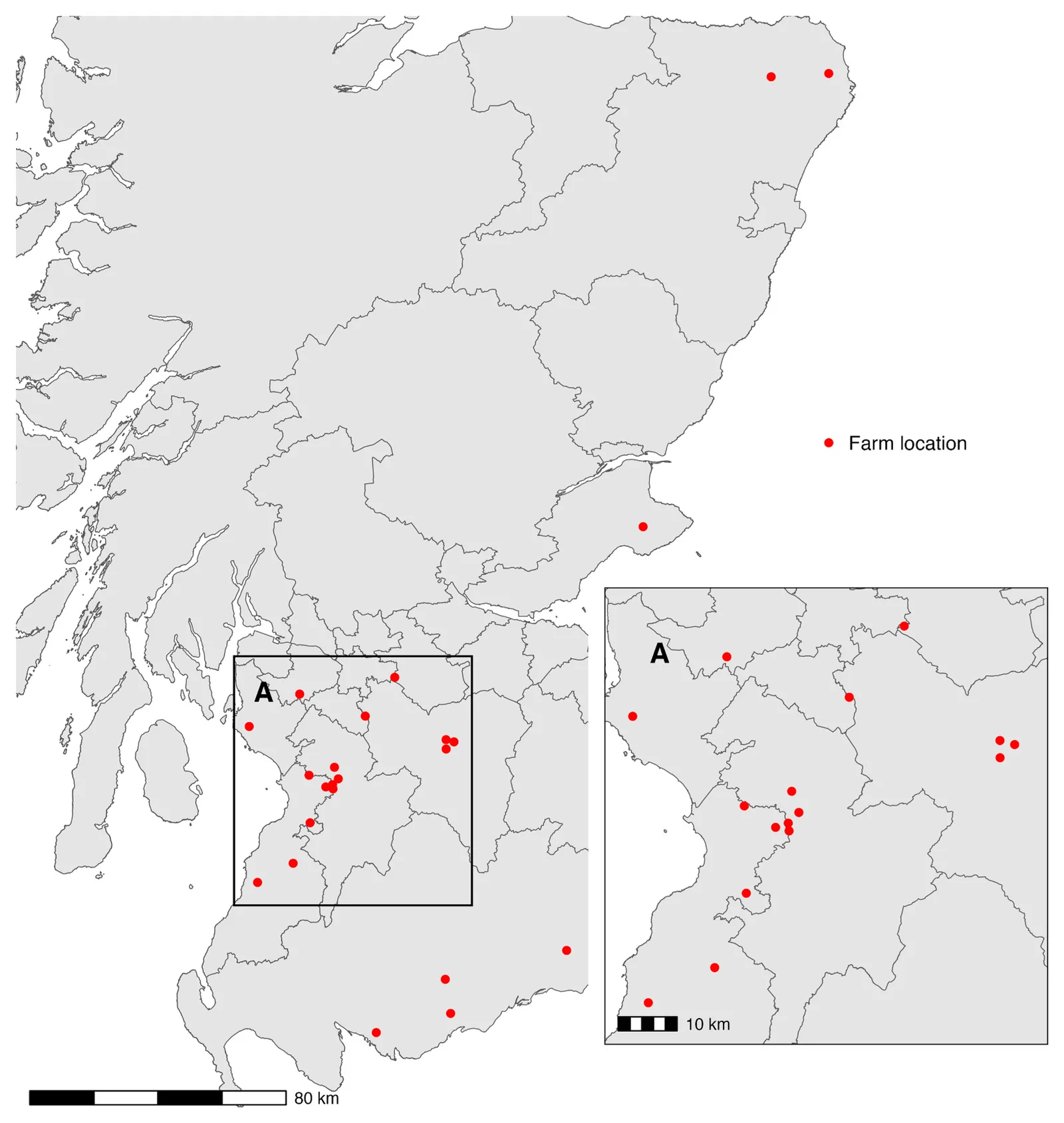
Map of the study area and locations in mainland Scotland. The map shows the distribution and approximate locations of dairy farms participating in the study across mainland Scotland. (A) represents the zoomed inset of Southwest Scotland, where many of the farms were located. Freshly voided faecal samples were collected from May to November 2022 at the sites indicated. Shapefiles for Scotland were obtained from Boundaries Scotland (OS_BL_ScottishLocalAuthority).

Using a non-interventionist approach, this longitudinal study was designed to monitor faecal egg counts (FEC) and estimate GIN species prevalence in first-grazing-season (FGS) dairy calves at monthly intervals. Simple inclusion criteria enabled the recruitment of a broad range of farms, requiring minimal input from participants to minimise burdens and promote participation. To participate, farms had to have turned out a minimum of 30 first-grazing season dairy calves before July 2022. They were also required to complete an animal husbandry questionnaire and allow for faecal sample collection on a monthly basis. No advice regarding anthelmintic treatments was provided, and farms were encouraged to manage animals as they normally would; FEC data were provided promptly, and participants could act on this information with guidance from their veterinarian. Twenty-three dairy farms completed an entire grazing season of sampling, of which four were organic and 19 were non-organic, located in Southwest, Central Scotland or Aberdeenshire (Fig. 1.). Farms were recruited through outreach through relevant stakeholder groups, milk suppliers, and private veterinary practices.

### 2.2 Sample collection and faecal egg counts

Sample collections began six weeks post-turnout of the calves to pasture on each farm and were repeated approximately every four weeks until the end of the grazing season. Each farm was visited 4 – 7 times between May and October 2022, resulting in a total of 131 sampling time points. At each farm visit, 15 individual fresh faecal samples were randomly collected directly from the pasture by surveying the field grazed by FGS dairy youngstock. Only samples identifiable as fresh (i.e., glossy and warm to the touch) were collected, and fresh faeces with similar appearance and consistency that were less than 3 m apart were ignored to minimise the possibility of repeat-sampling the same individual. Faeces were collected from the centre of the pat and transferred to 120 ML sterile specimen containers, sealed, and transported to the University of Glasgow on the same day. In total, 1967 faecal samples were collected over the 2022 grazing season. Aliquots for the FEC were prepared on arrival and stored at 4 ° C, while samples for coprocultures were stored at room temperature and processed within 24 h of collection.

A FEC was conducted on every individual sample collected using a modified, highly sensitive three-chamber McMaster technique (Paras et al., 2018) with a detection limit and multiplication factor of 8 eggs per gram (EPG). Briefly, 4 g of faeces were homogenised with water in a 1:10 faeces-to-water suspension. The suspension was then passed through a 250 μm sieve and further diluted to a 1:15 suspension by rinsing the sieve. A 15 ML aliquot was centrifuged at 1061 xg for 5 min, and the supernatant was removed. The pellet was resuspended in a saturated salt solution (sodium chloride) and inverted several times. Immediately after inversion, an aliquot was removed to fill a three-chamber McMaster slide. McMaster slides were read after waiting a minimum of five minutes, only counting eggs at least partially within the inner and outer gridlines. GIN eggs were identified morphologically and counted as strongyle-type or *Nematodirus* spp.

### 2.3 Coproculture and individual crude lysates

Pooled larval coprocultures from each visit were set up by hand-mixing approximately equal volumes of faeces with vermiculite to form a well-aerated, uniform paste-like consistency and incubated at 27 C for 14 days. Cultures were sprayed with water during incubation to ensure adequate moisture for L_3_ development. After the incubation period, the larvae were harvested using a modified Baermann technique as described in Roberts and O’Sullivan, 1950; pooled aliquots of L_3_ in double-distilled H_2_O were stored at –80 ° C.

Crude individual worm lysates (n = 13,292) were produced from single strongyle larvae in 96-well plates using a modified proteinase K lysis reaction. Briefly, 100x volume solution of lysis buffer was made as follows: 1000 μl DirectPCR Lysis Reagent (Cell) (Viagen Biotech), 50 μl 1 M DTT (Invitrogen), and 10 μl Proteinase K (100 mg/ML) (Invitrogen) and 10 μl was aliquoted per well of a 96-well PCR plate. Using a stereo microscope, individual larvae were transferred into each well in a volume of < 1 μl. Lysates were then incubated at 60 °C for 2 h, followed by 85 °C for 45 min to denature the Proteinase K. Crude lysates were aliquoted in 1:20 dilutions using nuclease-free water.

### 2.4 Strongyle species identification

For each sampling time point, a minimum of 94 recovered larvae were identified to species or genus level by PCR using the ITS2 region. A multiplex PCR method (Bisset et al., 2014) was employed, using primers designed to amplify the strongyle ITS2 region and regions specific to GIN species of interest (see Supplementary File 1). Two reaction sets were employed; set one included primers targeting *Haemonchus spp*., *Os. ostertagi, C. oncophora, Oesophagostomum venulosum*, and *Trichostrongylus. axei*. Reaction set two targeted *Ostertagia leptospicularis, Teladorsagia circumcincta*, and *Trichostrongylus colubriformis* and used for strongyles not identified by reaction set one.

The multiplex PCRs were performed in 96-well plates with 94 individual worm lysates, one PCR-negative control (no genomic DNA template), and one lysis-negative control (lysate without larva). Eurofins Genomics sequenced the amplicons of the conserved ITS2 region of any strongyle not identified to the species level by either reaction set. Then, a BLASTn search was performed using a curated ITS2 ribosomal DNA database (Workentine et al., 2020) for identification. To confirm the accuracy of the primers employed, five positively identified GIN for each primer pair had their entire ITS-2 region amplified and sent for capillary sequencing. All samples were positively identified as the correct species, and primer HACOFD3 identified only *H. contortus. Haemonchus placei* has never been reported in the UK to date, and UK-wide Nemabiome datasets have exclusively identified *H. contortus* (Hogg et al., 2010; Jewell et al., 2024; McGregor, 2024); therefore, we have assigned all HACOFD3-positive GIN as *H. contortus*.

### 2.5 Management questionnaire design and analysis

A questionnaire (n= 36 questions) was developed to collect farm management data in three sections (details provided below) (Supplementary File 2).

(i) Farm demographics and key performance indicators. (Q1 – Q8)
(ii) General husbandry and pasture management practices. (Q9 – Q18)
(iii) Anthelmintic usage and decision-making. (Q19 – Q36)

The researcher completed the questionnaire through a semi-structured interview, allowing for free conversation guided by the questionnaire. The questionnaire was piloted with four volunteer farmers to sense-check the questions and ensure clarity.

### 2.6 Faecal egg count and multispecies abundance data analysis

All statistical analysis and visualisation were performed using R studio version 2024.12.1 + 563, and the code used to analyse raw data and generate figures is available from GitHub (https://github.com/pau1campbe11/Seasonal-patterns-of-FEC-and-GIN-composition).

## 3 Results

### 3.1 Farm profiles

The basic farm demographics and anthelmintic use information are summarised in Table 1. Twenty-six farms were initially recruited for this study, of which 23 completed the entire sample collection season. Of the 23 farms, 11 were dairy-only systems, nine were dairy and beef, one was dairy and sheep, and two were dairy, beef and sheep systems. Four organically certified farms participated in the study, two of which had a “cow-with-calf” policy where calves were kept at foot with the dam until weaning at 5–6 months of age as opposed to most dairy systems where calves are removed from the dams within the first 24 h and weaned at ~8 weeks of age or older. An extensive range of FGS calf group sizes were included, ranging from 29 to 105 individuals, with ages ranging from 3 to 11 months, of which 63–100 % were female.

Macrocyclic lactones were the most commonly used anthelmintic class during the study, with limited use of benzimidazoles and no reported use of levamisole (Table 1). Across all farms, there were a total of 25 anthelmintic treatments given during the study (including treatments given at turnout but excluding treatments at housing). Of these treatments, eight were ivermectin, six moxidectin (three were moxidectin long-acting injectable products), seven doramectin, and four fenbendazole. The most anthelmintic treatments administered in a single grazing season on an individual farm were three doses of ivermectin given by Farm 1. In contrast, four farms (three organic and one non-organic) did not treat any animals during the grazing season. There were three broad categories of treatment regimens employed by the farms in the study, which we define as follows:

– Neo-suppressive: treatment to limit the establishment of a parasitic infection and minimise pasture larval contamination
– Prophylactic: treatment of an at-risk group in anticipation of clinical or production-limiting parasitism based on previous management experience, but without the use of diagnostic indicators
– Tactical: treatment of a group based on an indicator of sub-clinical infection that may be production-limiting

All treatments using the long-acting moxidectin formulation were classed as neo-suppressive (n = 3, Farms 13, 14, 17). Four farms utilised a tactical treatment regimen where animals were treated after veterinary advice based on an FEC, or after an observed reduction in growth rates (Farms 3, 12, 23 and 25). The remaining 16 farms utilised a prophylactic regimen where animals were treated at a specific time point in anticipation of a reduction in growth rates or increased risk of parasitic bronchitis (*Dictyocaulus viviparus* infection). All anthelmintic treatments were administered at the group level, and no farm employed a targeted selective treatment strategy. All farms utilised pour-on formulations of ML, except for those using long-acting moxidectin products, which are administered by subcutaneous injection. All benzimidazole treatments were administered orally, and no farms utilised an intraruminal bolus.

Of the seven farms that indicated that they had changed anthelmintics during the previous five years, only one was concerned about ineffective treatment/resistance in GIN. Three farms indicated they had changed anthelmintic drugs based on veterinary advice as part of a planned rotation/herd health plan, and four were influenced by their milk buyers to reduce ML use (specifically long-acting moxidectin formulations). Effective quarantine was not routinely practised on any farm in this study: only two farms stated that they gave quarantine treatments to bought-in cattle, and both stated that they do not routinely treat every animal.

### 3.2 Pasture management

The majority of farms (18/23 farms) turn out calves to graze on the same pasture in spring every year, potentially creating grazing areas of “higher risk” for GIN infection. Of these, 78 % (14/18 farms) would re-graze the same individuals on the same pasture in the autumn. These pastures of potentially “higher risk” were also permanent pastures, not cropped or re-seeded in the previous five years on most farms (13/14). Over half (15/23) of farms employed a form of rotational grazing, of which 33 % (5/15) rotated after a set number of days, and the remaining farms regularly rotated based on grazing availability. Seventeen per cent (4/23) of farms set-stocked their calves for the majority of the grazing season, all of which changed pasture within the last 34 days of their respective grazing season. The remaining farms (4/23) changed pasture on an ad hoc basis, averaging two changes per season.

### 3.3 Farmer attitudes

Only one farm expressed concern about anthelmintic resistance on their own farm, and no farm had ever previously tested for anthelmintic efficacy using the faecal egg count reduction test (FECRT). Four farms routinely employed FECs as a decision-making tool. Eight farms were actively aiming to reduce the frequency of anthelmintic treatment or to minimise treatment frequency. Only 22 % of farms regarded GIN infections as of particular concern, but more (65 %) regarded lungworm infection as of particular concern.

### 3.4 Species composition of GIN communities in Scottish dairy calves

Faecal egg counts were performed using individual animal samples, and GIN species identification was performed using a multiplex PCR of L_3_ cultured from pooled faeces from each calf group. Overall, FECs remained low throughout the grazing season on all farms (0–480 EPG), with the FEC distribution summarised in Table 2. The majority of GIN eggs were Strongyle-type but with *Nematodirus spp*. eggs observed frequently on 22/23 farms and in 23 % of samples. The *Nematodirus spp*. FEC ranged from 0 to 32 EPG but only accounted for 5.2 % of the total GIN FEC in this study.

**Fig. 2:**
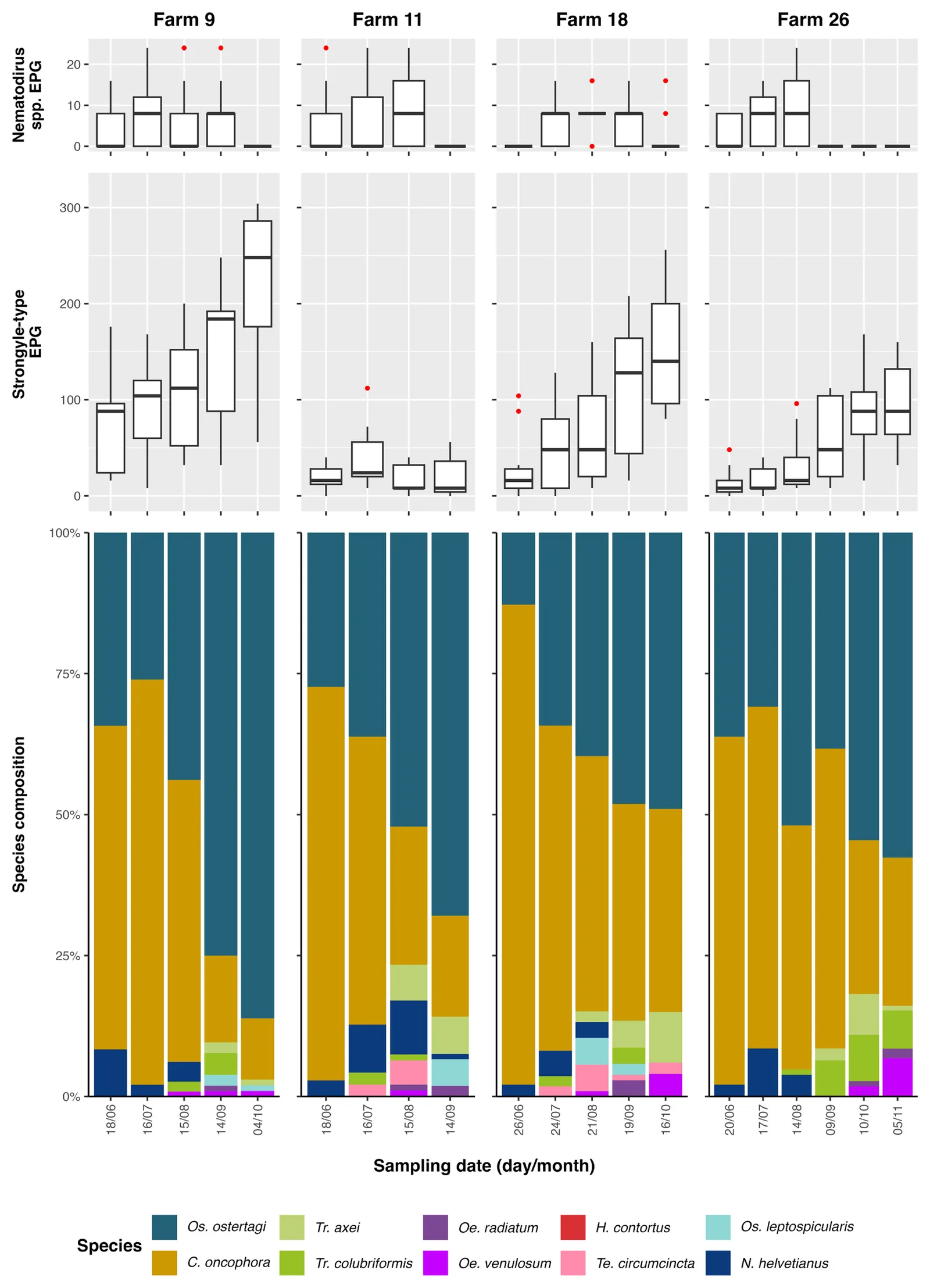
Relative species abundance and faecal egg counts of gastrointestinal nematode communities of FGS calves not treated with an anthelmintic during their grazing season. Species identity was assigned by ITS-2 rDNA multiplex PCR from group-level pools of L3 larvae harvested from coprocultures from each visit. A minimum of 94 L3 larvae were identified per pooled coproculture. For each farm and sampling timepoint, the upper boxplot indicates the *Nematodirus* spp. faecal egg count (FEC). The larger boxplot below represents the respective Strongyle FEC. Each red dot represents an outlier FEC. EPG = eggs per gram of faeces. The main bar chart in each panel shows the species composition of the larval cultures. For interpretation of the time points and references to colour in this figure legend, the reader is referred to the Web version of this article.

**Fig. 3:**
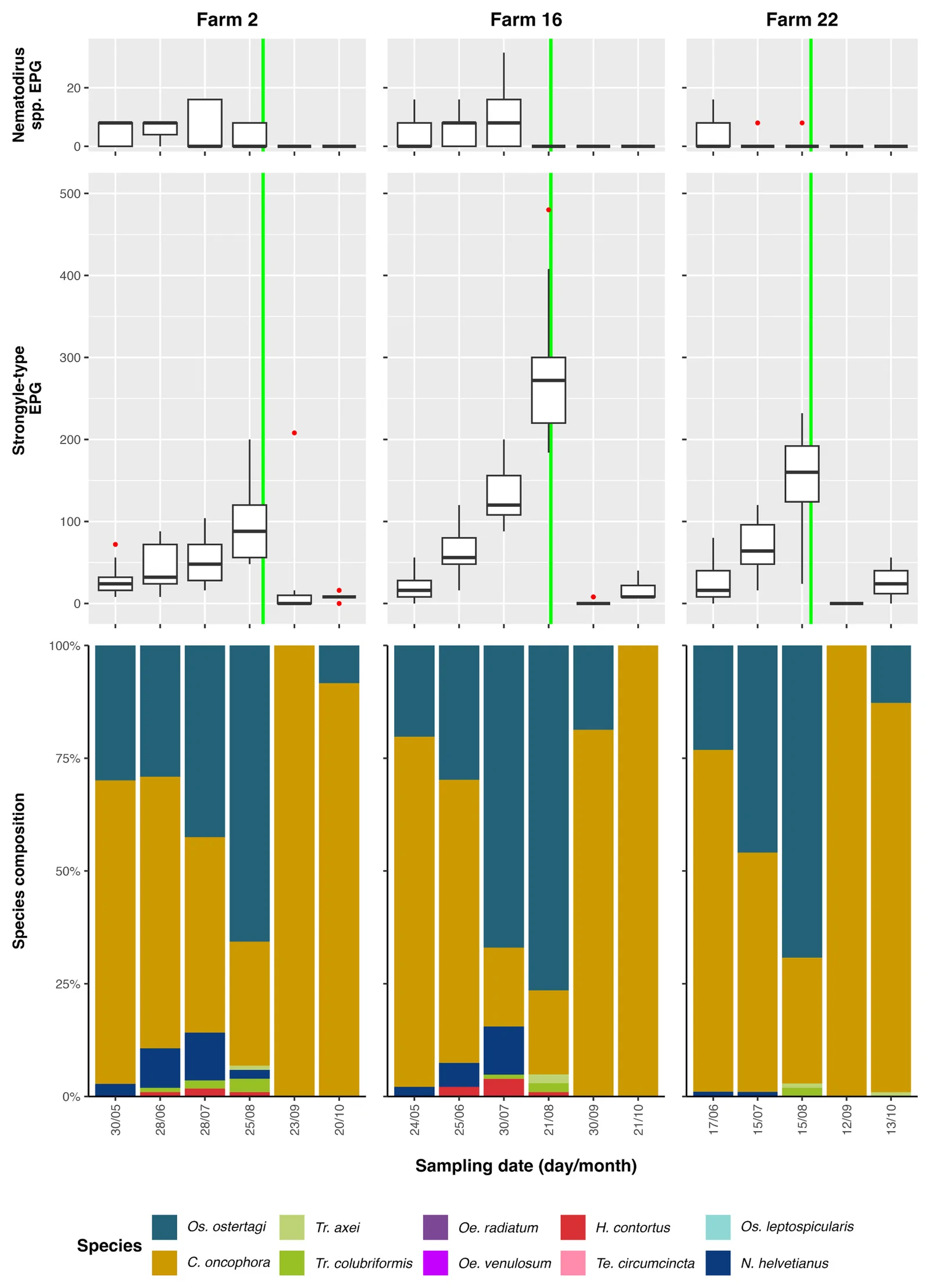
Relative species abundance and faecal egg counts of gastrointestinal nematode communities of FGS calves treated with a moxidectin product during their grazing season. Species identity was assigned by ITS-2 rDNA multiplex PCR from group-level pools of L3 larvae harvested from coprocultures from each visit. A minimum of 94 L3 larvae were identified per pooled coproculture. For each farm and sampling timepoint, the upper boxplot indicates the *Nematodirus* spp. faecal egg count (FEC). The larger boxplot below represents the respective Strongyle FEC. Each red dot represents an outlier FEC. The vertical green line represents when the moxidectin treatment was administered. EPG = eggs per gram of faeces. The main bar chart in each panel shows the species composition of the larval cultures. For interpretation of the time points and references to colour in this figure legend, the reader is referred to the Web version of this article.

**Fig. 4:**
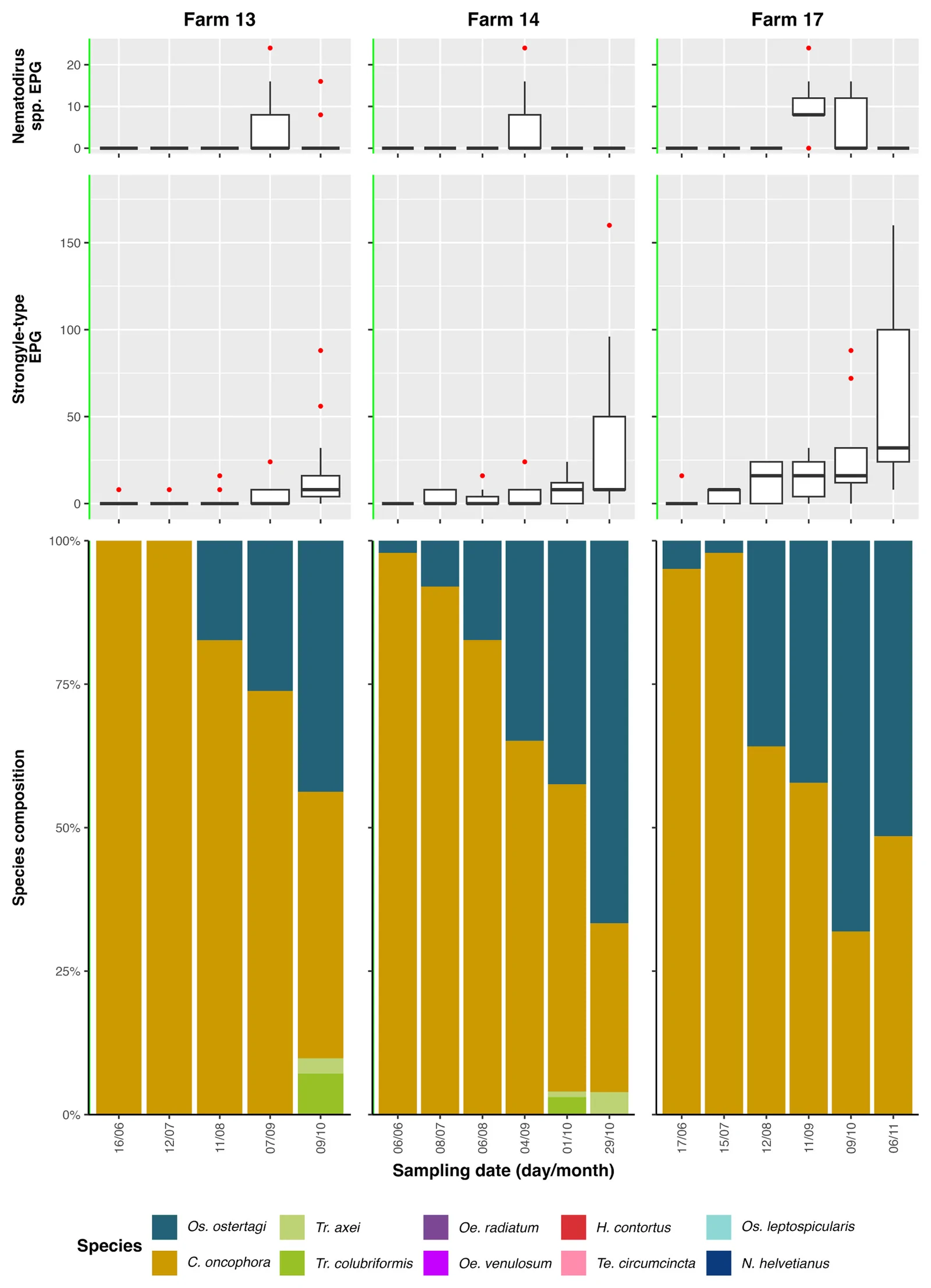
Relative species abundance and faecal egg counts of gastrointestinal nematode communities of FGS calves treated with a moxidectin long-acting product at turnout. Species identity was assigned by ITS-2 rDNA multiplex PCR from group-level pools of L3 larvae harvested from coprocultures from each visit. A minimum of 94 L3 larvae were identified per pooled coproculture. For each farm and sampling timepoint, the upper boxplot indicates the *Nematodirus* spp. faecal egg count (FEC). The larger boxplot below represents the respective Strongyle FEC. Each red dot represents an outlier FEC. The vertical green line represents when the moxidectin long-acting treatment was administered. EPG = eggs per gram of faeces. The main bar chart in each panel shows the species composition of the larval cultures. For interpretation of the time points and references to colour in this figure legend, the reader is referred to the Web version of this article.

**Fig. 5:**
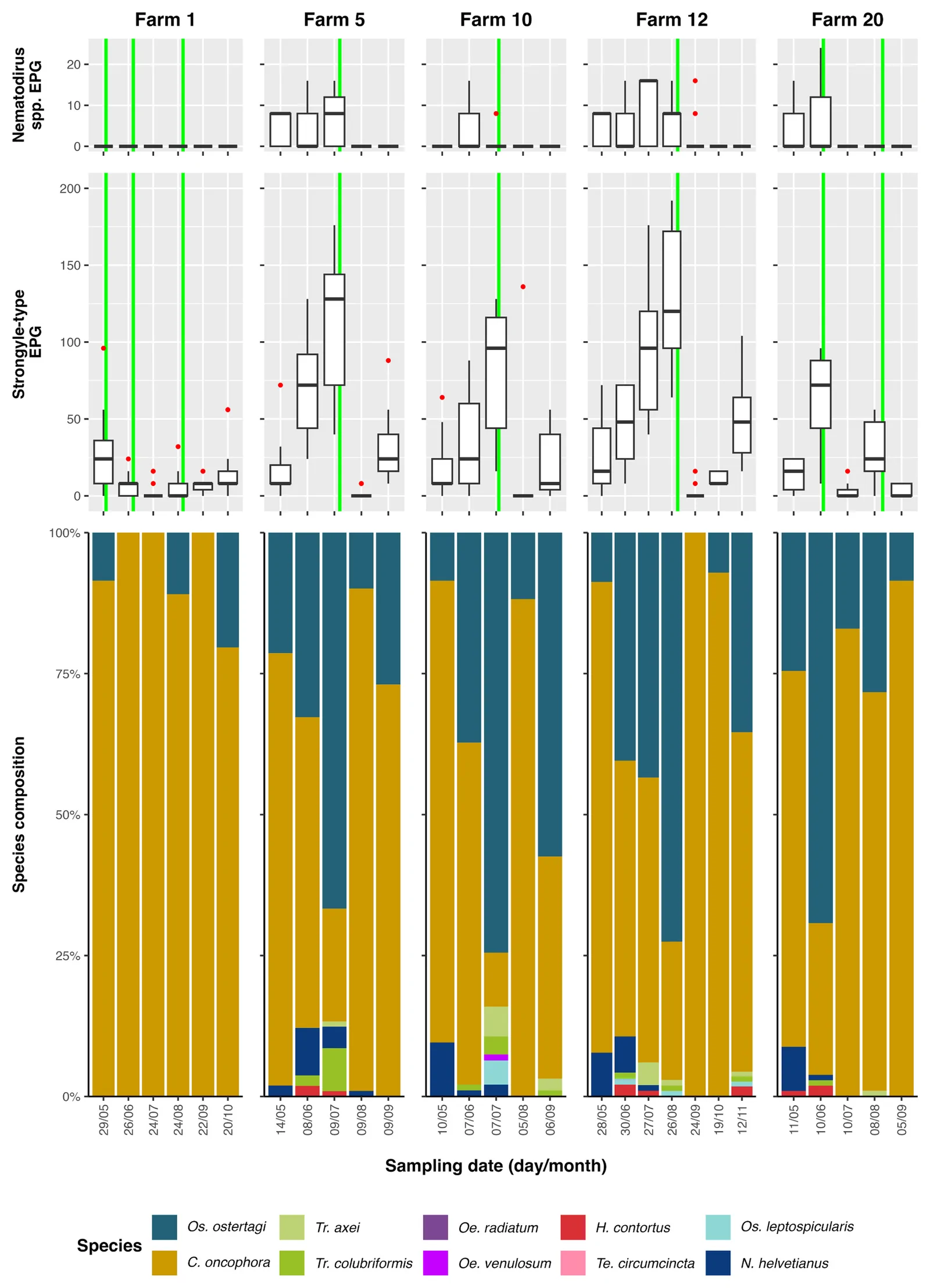
Relative species abundance and faecal egg counts of gastrointestinal nematode communities of FGS calves treated with an ivermectin product during their grazing season. Species identity was assigned by ITS-2 rDNA multiplex PCR from group-level pools of L3 larvae harvested from coprocultures from each visit. A minimum of 94 L3 larvae were identified per pooled coproculture. For each farm and sampling timepoint, the upper boxplot indicates the *Nematodirus* spp. faecal egg count (FEC). The larger boxplot below represents the respective Strongyle FEC. Each red dot represents an outlier FEC. The vertical green line represents when the ivermectin treatment was administered. EPG = eggs per gram of faeces. The main bar chart in each panel shows the species composition of the larval cultures. For interpretation of the time points and references to colour in this figure legend, the reader is referred to the Web version of this article.

**Fig. 6:**
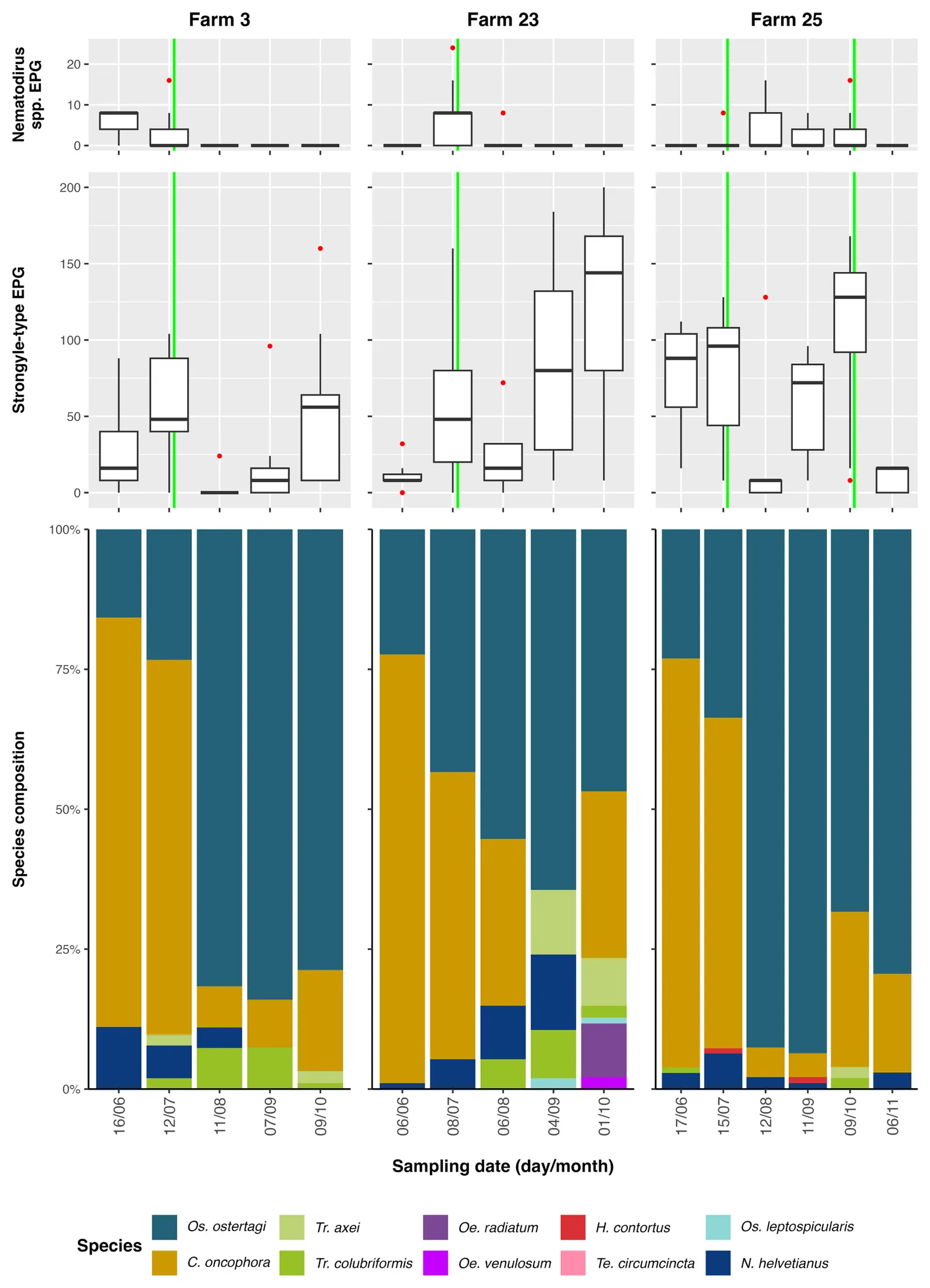
Relative species abundance and faecal egg counts of gastrointestinal nematode communities of FGS calves treated with fenbendazole product during their grazing season. Species identity was assigned by ITS-2 rDNA multiplex PCR from group-level pools of L3 larvae harvested from coprocultures from each visit. A minimum of 94 L3 larvae were identified per pooled coproculture. For each farm and sampling timepoint, the upper boxplot indicates the *Nematodirus* spp. faecal egg count (FEC). The larger box plot below represents the respective Strongyle FEC. Each red dot represents an outlier FEC. The vertical green line represents when the fenbendazole treatment was administered. EPG = eggs per gram of faeces. The main bar chart in each panel shows the species composition of the larval cultures. For interpretation of the time points and references to colour in this figure legend, the reader is referred to the Web version of this article.

**Fig. 7:**
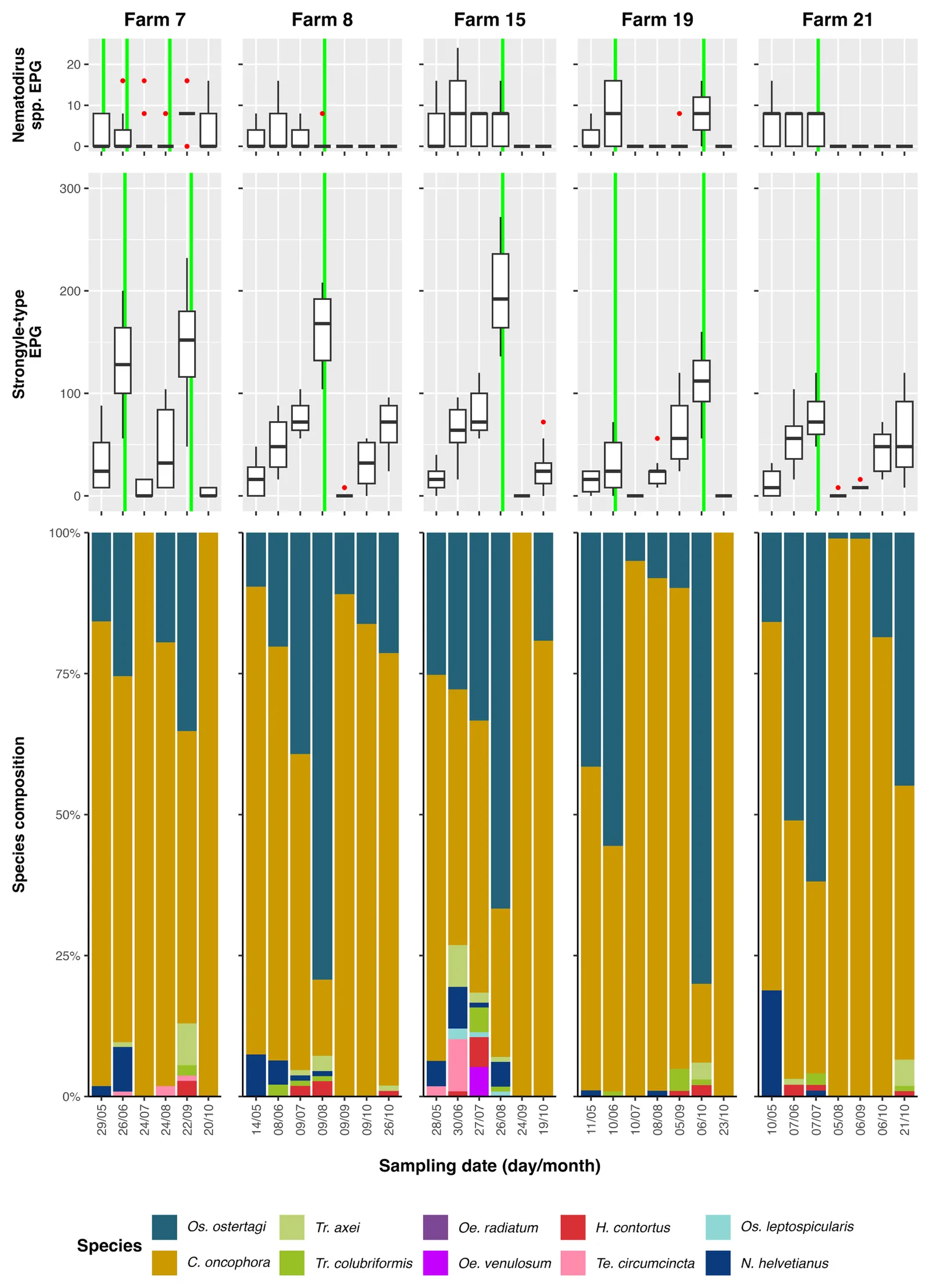
Relative species abundance and faecal egg counts of gastrointestinal nematode communities of FGS calves treated with a doramectin product during their grazing season. Species identity was assigned by ITS-2 rDNA multiplex PCR from group-level pools of L3 larvae harvested from coprocultures from each visit. A minimum of 94 L3 larvae were identified per pooled coproculture. For each farm and sampling timepoint, the upper boxplot indicates the *Nematodirus* spp. faecal egg count (FEC). The larger box plot below represents the respective Strongyle FEC. Each red dot represents an outlier FEC. The vertical green line represents when the doramectin treatment was administered. EPG = eggs per gram of faeces. The main bar chart in each panel shows the species composition of the larval cultures. For interpretation of the time points and references to colour in this figure legend, the reader is referred to the Web version of this article.

Despite differences in both the magnitude and timing of increasing FEC between each farm and variations among farms using similar treatment regimens and anthelmintics, clear trends and patterns were evident upon visual inspection (Figs. 2–7). The population dynamics of GIN infection throughout the grazing season are shown as individual farms grouped by anthelmintic product used. The efficacy of treatments could be clearly observed as decreases in FEC, but it was not calculated as the sampling intervals were not equivalent to those of an FECRT. These data illustrate that all prophylactic and strategic treatments were associated with dramatic decreases in FECs. On the farms that did not treat calves during the grazing season, a gradual increase in FECs was observed, except for Farm 11 (Fig. 2), where FECs were consistently very low and did not increase as the season progressed.

The occurrence of *Nematodirus* spp. in the FECs was transient and generally only present during the first three months of the grazing season. *Nematodirus* spp. were not observed during the period of protection provided by MOX-LA but did appear transiently in September on these farms. Except in samples taken during the periods of residual activity, the individual FECs often appeared over-dispersed throughout the dataset, the extent of which is highlighted by Farm 14 on the sixth consecutive timepoint (day 194), where the FEC had a mean of 34 and ranged from 0 to 168 epg (Fig. 4).

The composition of gastrointestinal nematode species present in pooled faecal samples was determined for all 131 sampling visits, with 13,297 individual larvae identified to species level; the observed species frequencies are summarised in Table 3. Ten gastrointestinal nematode species from seven genera were detected: *Os. ostertagi, C. oncophora, Tr. axei, Nematodirus helvetianus, Tr. colubriformis, Os. leptospicularis, Te. circumcincta, H. contortus, Oesophagostomum radiatum*, and *Oe. venulosum*. The two most prevalent species were *Os. ostertagi and C. oncophora* (p < 0.04), which were present on all farms and detected in 90.1 % and 99.2 % of the 131 pooled samples, respectively (Table 3). The next most prevalent species was *Tr. axei*, detected on 20/23 farms and in 30.5 % of pooled samples, showed no significant association with any herd demographic characteristic (>0.05). *Haemonchus contortus* was present on 47.8 % of farms and showed a marked regional distribution, primarily in South and East Ayrshire. *Os. leptospicularis, Te. circumcincta*, and *Oesophagostomum* spp. were present in up to seven farms and 10.7 % of samples and showed no significant association with any demographic characteristic (>0.05). The GIN community for each sample is shown in Figs. 2–7, grouped by the anthelmintic regimes.

The abundance of the two predominant GIN species, *Os. ostertagi* and *C. oncophora*, varied in proportion over time; in general, on each farm, *C. oncophora* was the most abundant species observed at the beginning of the grazing season, and as the season progressed, *Os. ostertagi* became more dominant. This pattern is most evident on the farms that did not treat during the grazing season (Fig. 2) and those that administered a fenbendazole product (Fig. 4). This dynamic, however, is disturbed when an anthelmintic treatment is given; in the case of the ML treatments, a dramatic increase in the proportion of *C. oncophora* L_3_ observed after treatment coinciding with a decrease in *Os. ostertagi* (Figs. 3–5 and 7). The opposite effect was observed after fenbendazole treatment (Fig. 6), where an increase in *Os. ostertagi* proportion and reductions of *C. oncophora* are observed. A resumption of this pattern of *Os. ostertagi* becoming progressively more abundant is clearly observed after the end of the period of residual anthelmintic activity on most farms.

## 4 Discussion

In this study, we investigated the seasonal patterns of FECs and nematode species composition in first-grazing season dairy calves on 23 farms in the northern temperate climate zone of Scotland. Understanding the infection patterns of GINs through the grazing season and the impact of anthelmintic treatments is crucial in supporting the development of evidence-based, sustainable parasite control strategies. Still, contemporary data on GIN species prevalence and infection intensities in UK cattle systems is lacking. Only one published study used a PCR platform to identify GIN (Roeber et al., 2017a), but this study did not collect any herd or farm characteristics. Most information from the UK is based on small-scale studies of one or two groups of cattle using morphological identification and the relatively insensitive McMaster technique with a sensitivity of 50 EPG. This study is in good agreement with these earlier studies, highlighting many similarities between morphological studies regarding GIN population dynamics over a grazing season and the effect of anthelmintic treatment (Armour, 1989; Kloosterman, 1971; Nansen et al., 1988; Steffan and Nansen, 1990). The identification of a wide range of less common and clinically significant genera such as *Trichostrongylus, Nematodirus* and *Oesophagostomum* spp. more frequently than previously reported, likely, however, reflects the greater accuracy and higher throughput of assigning species identity by PCR.

One of the major challenges of undertaking studies assessing GIN infections in cattle is the low FEC of individual cattle and, consequently, low recovery of larvae from faecal samples, particularly post-treatment and within the window of residual activity. To overcome this, a large pooling strategy was used to archive larvae collected during these periods of residual activity; this enabled the collection of significant numbers of larvae (>1000) from each sampling visit, even when the FEC were below the 8 EPG limit of detection. No clinical disease associated with GIN infection was reported on any farms during the study. However, Farm 10 experienced an outbreak of parasitic bronchitis, characterised by mild clinical signs caused by *D. viviparus* infection, which was diagnosed by Baermann analysis.

It should be noted that the estimates of the relative abundance of different GIN species based on cultured L_3_ can yield quite different results from postmortem identification of adult GIN populations in the GI tract. Roberts (1957) and Peterson (1957) found that faecal examination often overestimates the abundance of *C. oncophora* compared to postmortem results (Anderson et al., 1965; Ciordia et al., 1964; Rose, 1968), with these differences attributed to differences in fecundity and reproductive biology. Consequently, the use of FEC is more likely to better describe future pasture contamination and parasite challenges. Potential limitations arise from the methodology of pooling samples for culture regardless of individual FEC and the variations in the time between sampling points. The approach employed in this study reflects the real-world conditions regarding investigating GIN infections on commercial dairy farms, as well as the numerous confounding variables, including the broad range of management and treatment regimens that complicate comparisons between farms. Given these challenges, this study’s results and discussion have been primarily focused on analysing the observed general trends in FEC and L_3_ species composition.

Although FEC does not accurately reflect the actual adult worm burden of the host, they are useful determinant of pasture contamination and, hence, the future risk of clinical and sub-clinical disease. Mean group EPGs > 200 around eight weeks after turnout are shown to be predictive of clinical PGE later if calves remain on the same pasture (Shaw et al., 1998). Strongyle infections were observed in 76.5 % of samples with a mean FEC of 63 EPG, while only 2.5 % of FECs were greater or equal to 200 EPG, none occurred during the first eight weeks after turnout.

There were no apparent treatment failures during the study. Although anthelmintic efficacy cannot be assessed from the data, all treatments coincided with a dramatic decrease in FEC. Clear trends can be observed for each anthelmintic treatment regimen. For example, very low FECs were observed during the periods of residual activity of macrocyclic lactone products, and gradual increases in FEC were observed towards the end of these periods in the case of long-acting moxidectin. On farms that did not treat with an anthelmintic during the study, gradual increases in FEC were observed over the grazing season on all but one farm.

Ten GIN species were detected, and at the species level, species abundance, distribution, and temporal changes agree well with previous studies in temperate zones (Michel, 1969d; Pafčo et al., 2024; Roeber et al., 2017b). *C. oncophora* was the most abundant species during the beginning of the grazing season, but as the season progressed, the prevalence of *Os. ostertagi* increased and became the dominant species in many cases. This is unsurprising as these species are well known to be adapted to this climate. *C. oncophora* was also the predominant species during the first few months after turnout, correlating well with data from Kloosterman, A., 1971. Several species primarily found in sheep were also observed: *Te. circumcincta* and *H. contortus*, but at low levels, as well as *Os. leptospicularis*, a GIN primarily of wild deer (Lyons et al., 2024).The generalist GIN species *Tr. axei* and *Tr. colubriformis* were observed on the majority of farms (87 % and 91.3 %, respectively) but only present at a minority of time points (<36 %) either transiently or towards the end of the grazing season. The presence of *H. contortus*, primarily in Ayrshire, is noteworthy as a species that can infect a range of ruminant hosts. However, it is relatively uncommon in its preferred sheep hosts in this region (Sargison et al., 2007). No apparent correlation was observed between the GIN species found and co-grazing sheep, albeit with a relatively small sample size. Similar results, however, have been reported where a low prevalence of *H. contortus* was observed in Scottish cattle (McGregor, 2024). The clinical significance of many relatively uncommon species is uncertain and rarely detected by veterinary surveillance in the UK, and is rarely associated with parasitic gastroenteritis (Jewell et al., 2023). Although *H. contortus* and *Oesophagostomum* species are more prevalent and economically important in subtropical and tropical areas, climate change in the UK, with its warmer and more humid conditions, may lead to these species assuming greater clinical significance. It is also challenging to hypothesise specific reasons for the differing species proportions as numerous factors may influence the predominance of one species over another, including climate, farm management and the presence of anthelmintic resistance in one species over others.

Comparing the impact of MLs to BZs reveals the effect of the anthelmintic class on post-treatment population composition. *Cooperia* spp. are known to be dose-limiting for IVM and MOX (Benz and Ernst, 1979; Ranjan et al., 1992) and have been commonly observed pre-dominating after IVM treatment in Europe (Demeler et al., 2009; El-Abdellati et al., 2010; Familton et al., 2001); the data presented is consistent with this for all ML products. An opposite trend can be observed after fenbendazole treatment, where *Os. ostertagi* is the dominant species.

The farms in this study employed a range of different management practices, which likely significantly contributed to inter-herd variation. The use of macrocyclic lactone products is widespread in the UK, as reflected in the high proportion of farms in the study that use such products and the absence of any farm that has used levamisole. The high proportion of pour-on formulations likely reflects their ease of application, and interestingly, the only non-pour-on products used do not currently have an equivalent pour-on formulation. The two farms with a cow-with-calf policy are likely outliers and do not represent typical dairy systems. This is a very uncommon practice in the UK, and as calves are kept with the dam until weaning, it is likely more akin to a beef-suckler system.

Regarding the groups of FGS calves in this study, several conclusions can be drawn: clinical disease is unlikely to have occurred on these farms, and the impact of subclinical disease on production could not be estimated but was almost certainly occurring to a greater or lesser extent (Shaw et al., 1998). Again, this is complicated by the differences in pathogenicity and fecundity of GIN species. This is why species identification is an important factor and why GIN epidemiology should not be interpreted solely in terms of FEC. The impact of GIN infections could be interpreted as well-managed on all these farms, as FEC were relatively low throughout the study, and there were no outbreaks of clinical PGE. However, it is difficult to determine if the applications of anthelmintic products on these farms were always applied appropriately or if anthelmintic use could be reduced. Bovine lungworm infections are a concern in many systems and are treated with the same anthelmintic products. Outbreaks of clinical disease, however, were noted, but monitoring of sub-clinical infections was outside the scope of this study.

## 5 Conclusion

In summary, this longitudinal study has revealed the intra- and inter-herd differences in GIN species abundance and FEC intensities and the effect of anthelmintic treatments in FGS dairy calves. Species composition and FEC patterns varied in accordance with the expected patterns, yet varied considerably in relation to management and anthelmintic use. Ten species from eight genera were identified, and less common and clinically significant species were observed at greater frequency than expected. These new data represent the most comprehensive overview of the major GIN species across Scottish dairy farms to date. Substantial variance in the relative abundance of the two most predominant and clinically significant GIN species, *Os. ostertagi* and *C. oncophora*, between and within farms, were revealed, as well as the diversity and complexity of farm management systems. This study has provided updated data on the population dynamics of GIN infection using modern molecular techniques, serving as a baseline for future epidemiological studies.

## Supplementary Material

**Appendix A. Supporting information**

Supplementary data associated with this article can be found in the online version at doi:10.1016/j.vetpar.2025.110574.

Supplementary Files

## Figures and Tables

**Table 1:** Farm demographics summary.

Farm	Organic status	Dairy System	Calving pattern	No. of calves in study group	Age of calves in study group	Anthelmintic product used	No. of treatments	Anthelmintic total theoretical period of protection from *Os. ostertagi* infection
1	Non-organic	Dairy only	Spring-block	59	Spring-born	IVM	3	42
2	Non-organic	Dairy, beef, & sheep	AYR	78	Spring-born	MOX	1	35
3	Non-organic	Dairy only	Autumn block	79	Spring-born	FBZ	1	0
5	Non-organic	Dairy & beef	Spring block	105	Spring-born	IVM	1	21
7	Non-organic	Dairy & beef	Spring block	80	Spring-born	DOR	2	70
8	Non-organic	Dairy only	AYR	49	Spring-born	DOR	1	35
9	Non-organic	Dairy only	Dual-block	72	Autumn-born	None	0	0
10	Non-organic	Dairy only	AYR	43	Spring-born	IVM	1	21
11	Organic	Dairy & sheep^†^	Dual block	36	Spring-born	None	0	0
12	Non-organic	Dairy only	Dual block	53	Spring-born	IVM	1	21
13	Non-organic	Dairy & beef	AYR	48	Spring-born	MOX LA	1	120
14	Non-organic	Dairy & beef	AYR	70	Spring-born	MOX LA	1	119
15	Non-organic	Dairy & beef	AYR	63	Spring-born	DOR	1	35
16	Non-organic	Dairy & beef	AYR	91	Spring-born	MOX	1	35
17	Non-organic	Dairy & beef	AYR	39	Spring-born	MOX LA	1	120
18	Organic	Dairy, beef, & sheep	Dual block	39	Autumn-born	None	0	0
19	Non-organic	Dairy & beef	AYR	42	Autumn-born	DOR	2	70
20	Non-organic	Dairy only	AYR	54	Autumn-born	IVM	2	21
21	Non-organic	Dairy only	Spring block	89	Spring-born	DOR	1	35
22	Non-organic	Dairy & beef	AYR	70	Autumn-born	MOX	1	35
23	Organic	Dairy only	AYR	76	Spring-born	FBZ	2	0
25	Non-organic	Dairy only	Dual-block	114	Spring-born	FBZ	1	0
26	Organic	Dairy only^†^	Dual block	29	Spring-born	None	0	0

† Cow-and-calf policy where calves were reared with the dam until 5–6 months of age AYR: All-year-round, DOR: Doramectin, FBZ: Fenbendazole, IVM: Ivermectin, MOX: Moxidectin, MOX LA: Moxidectin long-acting formulation

**Table 2:** Frequency of occurrence of Strongyle and *Nematodirus* spp. type eggs in faecal egg counts (n = 1967).

Strongyle-type			*Nematodirus* spp. -type		
FEC	Frequency	Proportion of all samples (%)	FEC	Frequency	Proportion of all sample (%)
**0**	463	23.5	**0**	1492	75.9
**8–56**	939	47.7	**8**	332	16.9
**64–104**	309	15.7	**16**	117	5.9
**104–152**	124	6.3	**24**	24	1.2
**152–200**	83	4.2	**32**	2	0.1
**200–304**	41	2.1			
**304 +**	8	0.4			

**Table 3:** Frequency of occurrence and proportion of gastrointestinal nematode species in pooled cultures (n = 131) and at individual farm level (n = 23 farms).

	Pooled samples (n = 131)			Farm (n = 23)	
Species	Frequency of occurrence No. samples = n (%)		Inter-sample range (%)	Frequency of occurrence No. farms = n (%)	
*Ostertagia ostertagi*	118	(90.1)	0–93.6	23	(100)
*Cooperia oncophora*	130	(99.2)	0–100	23	(100)
*Trichostrongylus axei*	40	(30.5)	0–11.5	20	(87)
*Nematodirus helvetianus*	33	(25.2)	0–18.8	19	(82.6)
*Trichostrongylus colubriformis*	47	(35.9)	0–8.7	21	(91.3)
*Ostertagia leptospicularis*	14	(10.7)	0–4.7	7	(30.4)
*Teladorsagia circumcincta*	11	(8.4)	0–9.3	4	(17.4)
*Haemonchus contortus*	26	(19.8)	0–5.3	11	(47.8)
*Oesophagostomum radiatum*	7	(5.4)	0–9.5	5	(21.4)
*Oesophagostomum venulosum*	11	(8.4)	0–6.8	7	(30.4)
