# Supplementary material for: Seasonal patterns of faecal egg counts and gastrointestinal nematode species composition in Scottish dairy calves
Source: Vet Parasitol. Author manuscript; Available in PMC 2026 Sep 4. (PMC7619441; doi:10.1016/j.vetpar.2025.110574)
Supplement: Supplementary file 1 [file EMS218098-supplement-Supplementary_Files.zip › 1-s2.0-S0304401725001852-mmc1.docx]

**Supplementary file 1**

Primer sequences for ITS2 region used for gastrointestinal nematode identification, as developed by Bisset et al., 2014.

| **Target species/genus** | **Primer name** | **Sequence (5′–3’)** | **Melting temperature (T_m_)** | **Expected amplicon size (bp)** |
| --- | --- | --- | --- | --- |
| ITS2 Generic | ITS2GF | CACGAATTGCAGACGCTTAG | 54^◦^C | 370-398 |
|  | ITS2GR | GCTAAATGATATGCTTAAGTTCAGC | 54^◦^C |  |
| *Cooperia oncophora* | COONRV1 | CTATAACGGGATTTGTCAAAACAGA | 53^◦^C | 173 |
| *Haemonchus spp.* | HACOFD3 | CATGTATGGCGACGATGTTCTT | 55^◦^C | 90 |
| *Oesophagostomum venulosum* | OEVERV2 | CGACTACGGTTGTCTCATTTCA | 54^◦^C | 323/327/329 |
| *Ostertagia leptospicularis* | OSL3FD2 | CATGCAACATAACGTTAACATAATG | 52^◦^C | 196 |
| *Ostertagia ostertagi* | OSOSRV1 | CAATGTTAACGTCATGTTGCATTTCA | 55^◦^C | 207 |
| *Teladorsagia circumcincta* | TECIFD3 | GTACATTCAAATAGTAGCAATACGC | 53^◦^C | 295 |
| *Trichostrongylus axei* | TRAXFD1 | CAAATATTGTGATAATTCCCATTTTAGTTT | 53^◦^C | 236 |
| *Trichostrongylus colubriformis* | TRCORV1 | ACATCATACAGGAACATTAATGTCA | 52^◦^C | 232 |
| The first four letters of each primer name indicate the target species: OSOS = *Ostertagia ostertagi*. FD denotes forward primer  RV denotes reverse primer | | | | |
