# Supplementary material for: Seasonal patterns of faecal egg counts and gastrointestinal nematode species composition in Scottish dairy calves
Source: Vet Parasitol. Author manuscript; Available in PMC 2026 Sep 4. (PMC7619441; doi:10.1016/j.vetpar.2025.110574)
Supplement: Supplementary file 1 [file EMS218098-supplement-Supplementary_Files.zip › 1-s2.0-S0304401725001852-mmc2.docx]

**Supplementary file 2**

**Farm management questionnaire completed through a semi-structured interview.**

**Farm Management Questionnaire: University of Glasgow Dairy Worms Project – Sampling Summer 2022**

**Section 1: Milk Recording**

1. Does your farm milk record?

☐ Yes

☐ No

2. If yes, could we have access to your CIS/NMR records?

☐ Yes

☐ Vet login

☐ Farmer login

☐ No

**Section 2: Anthelmintic Treatments**

Section one refers only to the group of first grazing season calves that we will be sampling from this summer.

3. If your first grazing season calves have received an anthelmintic (wormer) treatment. What have they received and when?

| Product | When | Number of animals |
| --- | --- | --- |
|  |  |  |
|  |  |  |
|  |  |  |

.

4. When do you next plan to treat these calves for worms and with what products?

| Product | When |
| --- | --- |
|  |  |
|  |  |
|  |  |

.

**Section 3: General Farm & Farm Management**

5. How many years has your farm held organic status for?

_____________ Years

☐ Not applicable

6. What is your current herd size? Please fill in how many animals of each age range do you have.
a. All milking cows	_____________
b. Heifers 12 months to 1st calving	_____________
c. Weaned calves (up to 12 months)	_____________
d. Unweaned calves	_____________
e. Bulls	_____________

7. Do you have any beef cattle or sheep on the farm?

☐ Beef

☐ Sheep

☐ Neither

If yes, are the animals:

a. Only there for winter grazing?	☐ Beef	☐ Sheep
b. Always on farm	☐ Beef	☐ Sheep

8. Within the last 12 months, how many animals have been bought in?
a. Cows	_____________
b. Calves	_____________
c. Heifers	_____________
d. Bulls	_____________
e. ☐ No animals bought in

9. At what age do you aim to calve your heifers?

_____________ Months

10. What is your average milk yield (L/Cow/Annum)?

_____________

11. How many first grazing season calves did you turn out to pasture last year?

_____________

12. What were your first grazing season calf’s turnout and housing dates last year?
a. Turned out	_____________
b. Housed	_____________

13. When does your herd calve? Please fill in how many cows calved in each month of the past 12 months.

| Month | March | Feb | Jan | Dec | Nov | Oct | Sept | Aug | July | June | May | April |
| --- | --- | --- | --- | --- | --- | --- | --- | --- | --- | --- | --- | --- |
| Number Calved |  |  |  |  |  |  |  |  |  |  |  |  |

14. How are your first grazing season calves grazed?
a. What pasture types is used?

☐ Permanent pasture

☐ Temp leys

b. What pasture management system is used?

☐ Mob / Strip grazing

☐ Rotational grazing

☐ Set stocked

15. Do you turn out first grazing season calves onto the same pasture every year?

☐ Yes

☐ No

16. Do you move your first grazing calves to different pasture during the summer?

☐ Yes

☐ No

If yes, please tell us when and where?

________________________________________________________________________________________________________________________________________________________________

________________________________________________________________________________________________________________________________________________________________

17. What animals have been grazed on the first grazing season calves’ pasture in the last year?

________________________________________________________________________________________________________________________________________________________________

18. Complete the table by answering the following questions and marking it on the table?
a. Date on which you turned out calves to pasture
b. Number of calves turned out on these dates
c. Dates you wormed your calves
d. Type of wormer was used on these dates
e. Number of calves treated on these dates
f. Dates the group of the first turned-out calves changed pasture

| QUESTION | JAN | | | | FEB | | | | MAR | | | | APR | | | | MAY | | | | JUN | | | | JUL | | | | AUG | | | | SEP | | | | OCT | | | | NOV | | | | DEC | | | |
| --- | --- | --- | --- | --- | --- | --- | --- | --- | --- | --- | --- | --- | --- | --- | --- | --- | --- | --- | --- | --- | --- | --- | --- | --- | --- | --- | --- | --- | --- | --- | --- | --- | --- | --- | --- | --- | --- | --- | --- | --- | --- | --- | --- | --- | --- | --- | --- | --- |
| a |  |  |  |  |  |  |  |  |  |  |  |  |  |  |  |  |  |  |  |  |  |  |  |  |  |  |  |  |  |  |  |  |  |  |  |  |  |  |  |  |  |  |  |  |  |  |  |  |
| b |  |  |  |  |  |  |  |  |  |  |  |  |  |  |  |  |  |  |  |  |  |  |  |  |  |  |  |  |  |  |  |  |  |  |  |  |  |  |  |  |  |  |  |  |  |  |  |  |
| c |  |  |  |  |  |  |  |  |  |  |  |  |  |  |  |  |  |  |  |  |  |  |  |  |  |  |  |  |  |  |  |  |  |  |  |  |  |  |  |  |  |  |  |  |  |  |  |  |
| d |  |  |  |  |  |  |  |  |  |  |  |  |  |  |  |  |  |  |  |  |  |  |  |  |  |  |  |  |  |  |  |  |  |  |  |  |  |  |  |  |  |  |  |  |  |  |  |  |
| e |  |  |  |  |  |  |  |  |  |  |  |  |  |  |  |  |  |  |  |  |  |  |  |  |  |  |  |  |  |  |  |  |  |  |  |  |  |  |  |  |  |  |  |  |  |  |  |  |
| f |  |  |  |  |  |  |  |  |  |  |  |  |  |  |  |  |  |  |  |  |  |  |  |  |  |  |  |  |  |  |  |  |  |  |  |  |  |  |  |  |  |  |  |  |  |  |  |  |

**Section 3: Wormer (Anthelmintic) Use**

19. How do you determine how much wormer (anthelmintic product) to use?

☐ According to estimated weight of each animal

☐ According to estimated average weight of the group

☐ According to estimated weight of the heaviest animal in group

☐ Weigh individual animals and treat accordingly

20. Do you worm bought in animals on arrival?

☐ Yes

☐ No

If yes, what do you use?	_______________________________________________

21. Are all bought in animals kept separate (isolated) from the herd on arrival?

☐ Yes

☐ No

22. Has there been any reports of resistance to wormers (anthelmintic products) in your herd?

☐ Yes

☐ No

a. If yes, what product was the resistance to?	_____________
b. What was the resistant worm? (If known)	_____________
c. How was it diagnosed?

☐ Product had no or little effect

☐ Diagnosed by lab

☐ Other	_____________

23. Are you currently taking any measures to reduce the amount of wormer (anthelmintic drugs) used on your farm?

☐ Yes

☐ No

If yes, please specify what measures

________________________________________________________________________________________________________________________________________________________________________________________________________________________________________________________________________________________________________________________________________________________________________________________________________________________________________________________________________________________________

24. Do you or your vet conduct regular faecal egg counts on your farm?

☐ Yes

☐ No

25. When and why do you usually treat your first grazing season calves with an anthelmintic (wormer) product and what with?

________________________________________________________________________________________________________________________________________________________________________________________________________________________________________________________________________________________________________________________________________________________________________________________________________________________________________________________­­­­­­­­­­­­­­­­­­­­­­­________________________________________

26. Did you treat individual first grazing season calves due to a suspected worm problem in 2021?

☐ Yes

☐ No

a. How many animals?	_____________
b. What product did you use?	_____________
c. Why did you treat?	_____________

27. Did you treat all first grazing season calves due to a suspected worm problem in 2021?

☐ Yes

☐ No

a. When did you treat?	_____________
b. What product did you use?	_____________
c. Why did you treat?	_____________

28. What anthelmintic products have you given to your first grazing season calves up to the last 5 years?

| Year | Products used |
| --- | --- |
| 2021 | 1. |
|  | 2. |
|  | 3. |
|  | |
| 2020 | 1. |
|  | 2. |
|  | 3. |
|  | |
| 2019 | 1. |
|  | 2. |
|  | 3. |
|  | |
| 2018 | 1. |
|  | 2. |
|  | 3. |
|  | |
| 2017 | 1. |
|  | 2. |
|  | 3. |

29. Are there any parasites you believe to be a problem for your herd? (e.g., lungworm, lice)

________________________________________________________________________________________________________________________________________________________________________________________________________________________________________________
